# *In Vitro* Anthelmintic Activity of Saponins Derived from *Medicago* spp. Plants against Donkey Gastrointestinal Nematodes

**DOI:** 10.3390/vetsci6020035

**Published:** 2019-03-29

**Authors:** Michela Maestrini, Aldo Tava, Simone Mancini, Federica Salari, Stefania Perrucci

**Affiliations:** 1 Department of Veterinary Sciences- University of Pisa, Viale delle Piagge n. 2, 56124 Pisa, Italy; michela.maestrini@phd.unipi.it (M.M.); simone.mancini@for.unipi.it (S.M.); federica.salari@unipi.it (F.S.); 2 CREA-ZA Research Center for Animal Production and Aquaculture, Viale Piacenza 29, 26900 Lodi, Italy; aldo.tava@crea.gov.it

**Keywords:** donkey, gastrointestinal nematodes, saponins, prosapogenins, *Medicago* spp. plants, *in vitro* anthelmintic activity

## Abstract

With the aim to find new effective natural compounds for the control of nematodes, the *in vitro* anthelminthic properties of purified 1% saponins showing different chemical compositions and derived from *Medicago sativa* (MS), *Medicago arborea* (MA), *Medicago polymorpha* cultivar ‘Santiago’ (MPS), *M. polymorpha* cultivar ‘Anglona’ (MPA), and 1% prosapogenins from *M. sativa* (MSp), were evaluated and compared. As a source of nematode eggs, pooled fresh fecal samples taken from dairy donkeys naturally infected by gastrointestinal nematodes were used. From fecal samples, eggs were recovered, suspended in deionized water, and used immediately in the bioassay (egg hatch test). The activity of the tested compounds was compared to positive (0.1% thiabendazole) and negative (deionized water and 1% DMSO) controls. All experiments were repeated in triplicate and the obtained data were statistically analyzed. All the tested plant compounds caused a significant (*p* < 0.05) inhibition of nematode egg hatching (>80%). Moreover, all saponins and prosapogenins showed *in vitro* anthelmintic properties statistically comparable to that of the reference drug (*p* < 0.05), except for MPS extract. Obtained results showed that the different *Medicago* saponins evaluated in this study possess high anthelmintic properties against gastrointestinal nematodes of dairy donkeys, although to a different extent depending on their composition.

## 1. Introduction

In recent years, the interest in the welfare and diseases of donkeys has considerably increased in Europe, mainly due to the popularity gained by donkey milk for human consumption and for the cosmetic industry [1]. Among pathogens of equids, gastrointestinal nematodes (GIN) are frequently associated with the onset of disease, the expiry of physical conditions, and the decline in productive and reproductive performances [2]. These parasites, especially cyathostomins, are currently included among the most frequent and pathogenic nematodes of donkeys in Europe [2]. 

In equids, the control of gastrointestinal nematodes is mainly based on the use of synthetic drugs, such as macrocyclic lactones and benzimidazoles [3]. However, the use of drugs for the control of GIN that can be excreted in milk or that can persist in treated animals is limited in dairy donkey farms [4,5,6], thus there is a need to develop new and alternative control methods.

Moreover, drug resistance developed by these parasites is widespread worldwide and a great problem in horse farming [2,7]. Therefore, various alternative methods for the control of gastrointestinal nematodes have been extensively studied in horse husbandry, such as pasture management [8], the use of nematophagous fungi [8], and of plant-derived compounds [9]. The latter approach is considered particularly promising for the control of gastrointestinal nematodes in farm animals due to the possibility of identifying new anthelmintic molecules, as well as directly using plants or plant-derived compounds for the formulation of active food supplements [10]. 

The egg hatch test (EHT) is a standard bioassay commonly used for the *in vitro* screening of the anthelmintic properties of natural compounds against gastrointestinal nematodes [11,12]. EHT is in fact considered a valid and useful test for the evaluation of the *in vitro* efficacy of drugs and one of the main *in vitro* methods used for the detection of benzimidazole resistance in nematode parasites [13,14]. This test consists in evaluating the ability of the tested substances to cause the inhibition of the development and hatching of GIN eggs [13,14].

Among plant-derived substances, saponins are considered as potential anthelmintic natural compounds [11,12]. Biological effects of saponins are normally ascribed to their specific interaction with cell membranes [15], causing changes in cell permeability. By affecting some cell membrane components, saponins induce formation of micelle-like aggregates that disrupt membrane functionality and cause lysis [16]. For nematodes, saponins have been associated with the formation of complexes with cellular membrane components present in different stages of nematode life cycle, leading to an increase in membrane permeability and causing these parasites to die [16,17]. 

Saponins are produced in many plant species, including mono- and dicotyledons. The Leguminosae family have been extensively investigated for their saponin content, and within this family of plants, *Medicago* species may represent a valuable rich source of bioactive saponins [15]. 

The genus *Medicago* includes several plant species, and some of them represent very important forage crop worldwide, such as alfalfa, *Medicago sativa* L., and burr medic, *Medicago polymorpha* L. [18]. Other species have an agronomic relevance in specific areas, such as tree medic, *Medicago arborea* L. in Mediterranean environments [19]. 

Saponins from *Medicago* species have shown to possess several biological activities; among these, they have been described to have hemolytic [17,20], antimicrobial [21,22], insecticidal [23], cytotoxic [24], allelopathic [15], and anthelmintic effects against some plant nematodes [25].

This study evaluated and compared the *in vitro* anthelmintic properties of purified saponins derived from different *Medicago* species characterized by different chemical compositions. For the evaluation of the *in vitro* anthelmintic properties of the tested saponins, EHT and donkey GIN eggs were used. 

## 2. Materials and Methods

### 2.1. Plant Material Extraction, Purification, and Characterization of Saponins

*Medicago* plants used in this study were grown at the Research Centre for Animal Production and Aquaculture (CREA-ZA, Lodi, Italy). Tops from *Medicago arborea* L., *Medicago polymorpha* cultivars ‘Santiago’, *M. polymorpha* cultivars ‘Anglona’, and *Medicago sativa* L. were utilized for saponin processing. Saponins were extracted and purified following general procedures previously reported [26,27]. In addition, saponins from *M. sativa* were subjected to basic hydrolysis [15] to extract the related prosapogenins, which were also evaluated in this study. All samples were dissolved in H2O-5% DMSO (2% *w/v*), and obtained solutions were used in the bioassay after further dilution at 1% in H_2_O-1% DMSO.

For characterization, purified mixtures of saponins obtained as whitish powders in pure grade (90−95%) were analyzed by thin layer chromatography (TLC), as previously described [28]. In addition, extracted and purified saponin mixtures were characterized for their qualitative and quantitative aglycone composition by gas chromatography (GC) and gas chromatography/mass spectrometry (GC/MS) analyses of derivative sapogenins obtained after acid hydrolysis, as already reported [28]. To obtain information on saponin composition (e.g., monodesmoside/bidesmoside compounds), the saponin mixtures were then analyzed by HPLC and the results compared with available data [15,26,27]. 

### 2.2. Nematode Egg Collection, Purification, and Suspension 

For nematode egg collection, individual fecal samples from donkeys of the Amiatina breed living in a dairy donkey farm located in central Italy (Grosseto, Italy) and found to be naturally infected by gastrointestinal nematodes (GIN) were used. For transportation to the laboratory, fecal samples were wrapped in an airtight plastic sleeve. Fecal microscopic analysis was performed using the McMaster technique with a sensitivity of 50 eggs per gram of feces (EPG) [20], and only samples positive for more than 1000 EPG were selected, pooled, and used to obtain GIN eggs. 

Recovery and suspension of GIN eggs were performed within 3 h of collection by using previously reported methods [13]. More specifically, 30 gr of feces were mixed with distilled water, sieved for clearing of gross organic debris, and centrifuged for 2 min at 300× *g* in 50 mL tubes. The sediment was collected, suspended in saturated NaCl solution (specific gravity 1.2), and centrifuged for 2 min at 130× *g*. The superficial layer containing the eggs was then collected, diluted in deionized water in 15 mL tubes, and washed by centrifugation for 2 min at 300× *g*. The collected sediment and the eggs contained therein were then inspected microscopically to evaluate if embryonation had begun, counted, diluted in deionized water to the final concentration of about 100 eggs/0.3 mL, and used immediately in the bioassay. 

Moreover, with the same pooled fecal samples used for obtaining GIN eggs, coprocultures were also performed [29] for the identification of GIN third stage larvae (L3) at the genus level. Fecal samples were cultured in an incubator at 25 °C for seven days and larvae were recovered by the Baermann technique [29] and identified according to previously reported keys and descriptions [30].

### 2.3. Evaluation of the In Vitro Anthelmintic Activity of Medicago Saponin Extracts

The *in vitro* anthelmintic activity of saponin and pro-sapogenin mixtures obtained from *Medicago* plants was evaluated using the egg hatch test (EHT). In the present study, the EHT was performed by using previously reported methods [13,14,31], with slight modifications. More specifically, in each well of 24-well flat-bottomed microplates, 0.8 mL of deionized water and 0.6 mL of egg suspension were placed so that each well contained about 200 eggs. Then, 0.6 mL/well of a single purified saponin extract from each *Medicago* plant species under evaluation were added. Wells containing the same amount of deionized water and egg suspension plus 0.6 mL of a 0.1% thiabendazole solution (Sigma Aldrich S.r.l, Milan, Italy) (0.1% TBZ) in 1% DMSO were used as positive controls. Two different negative controls were made, the first one by placing in each well 1.4 mL of deionized water and 0.6 mL of egg suspension, and the second one by placing in each well 0.8 mL of deionized water, 0.6 mL of egg suspension, and 0.6 mL of a 1% DMSO solution. Microplates were sealed to prevent evaporation and incubated for 48 h at 25 °C and 80% relative humidity. After this time, egg development was stopped by adding one or two drops of Lugol’s iodine solution, the number of eggs and first-stage larvae (L1) microscopically counted in each well, and the percentage of reduction calculated with the following formula:

(number of L1 in the control − number of L1 in the sample)/(number of L1 in the control) × 100.

All experiments were repeated in triplicate in three independent assays.

### 2.4. Statistical Analysis

Results obtained in EHT from negative and positive controls, and from the different *Medicago* saponin extracts under examination, were statistically analyzed and compared. Statistical analysis was performed using the Statistical Analysis System (SAS) program. To perform statistical analysis of the obtained data, a one-way ANOVA test with a 5% significance (*p* < 0.05) was used. Significant results were further tested with the Tukey post-hoc test (*p* < 0.05). 

### 2.5. Ethical Declaration

Approval for this study was obtained from the Ethical Committee on Animal Experimentation of the University of Pisa. In addition, authors declare that the work has been carried out in compliance with the European Directive regarding ethical use of animals and in adherence to a high standard of veterinary care.

## 3. Results

### 3.1. Composition of Medicago Saponins

**Figure 1:**
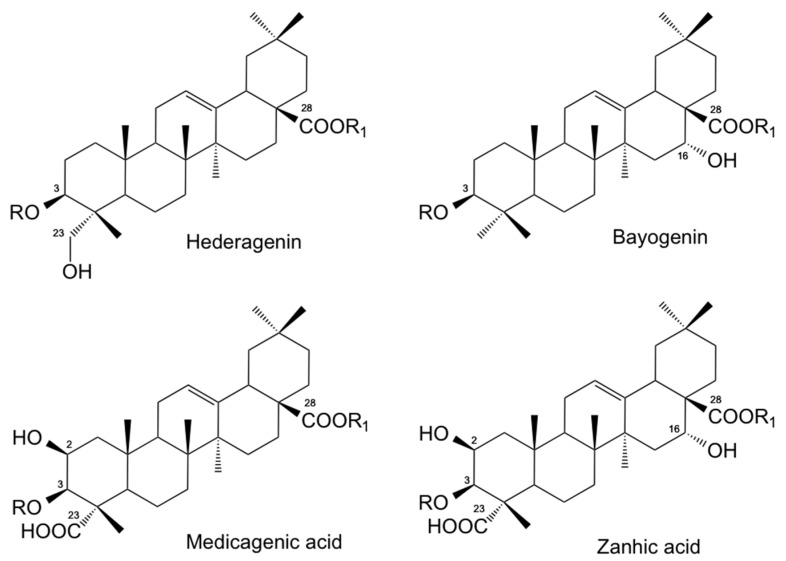
Chemical structure of the most abundant sapogenins (R=R1=H) detected in *Medicago* spp. plant extracts evaluated in this study for their *in vitro* anthelmintic activity against donkey gastrointestinal strongyles. Saponins: R=sugar or sugar chain, R1=H: monodesmosides. R=R1= sugar or sugar chain: bidesmosides.

The chemical structures of *Medicago* saponin extracts used in this study showed a compositional profile which differed according to the plant species. Based on the relative content of the dominant sapogenins after acid hydrolysis of the corresponding glycosides, saponins from *M. sativa* (MS) were characterized by a higher amount of medicagenic and zanhic acids (Figure 1), quoted as 47.2% and 23.4%, respectively. Medicagenic acid and zanhic acid were also the most abundant aglycones from saponins of *M. arborea* (MA), accounting for 28.1% and 44.7%, respectively. Hederagenin (Figure 1) was instead the dominant sapogenin in *M. polymorpha* cultivar ‘Santiago’ (MPS), representing 93.3% of the total aglycones, while echinocystic acid (88.1%) (Figure 1) was the dominant sapogenin detected in *M. polymorpha* cultivar ‘Anglona’ (MPA). From the HPLC analyses by comparison with authentic reference saponins previously identified in the *Medicago* spp., all the saponin mixtures here evaluated were mainly constituted by bidesmosidic type saponins (70−80%). *M. sativa* prosapogenins (MSp), obtained after basic hydrolysis of the corresponding saponins, were instead entirely made up by monodesmosides.

### 3.2. Evaluation of the In Vitro Anthelmintic Activity of Plant Extracts

**Figure 2:**
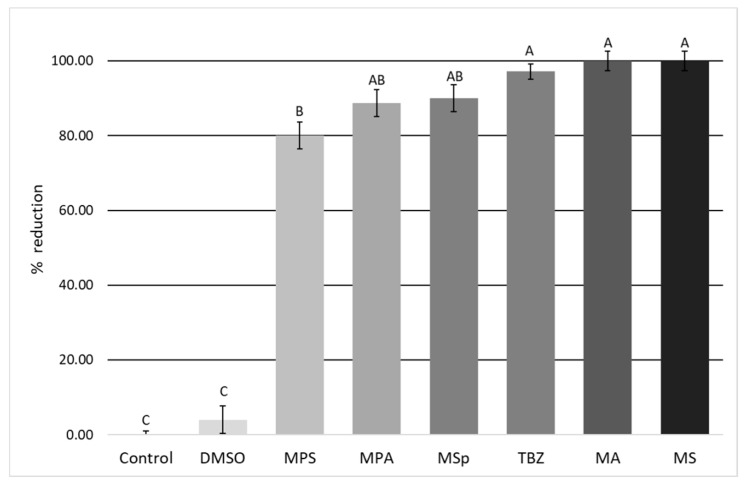
Egg hatching reductions (in %) observed for 1% prosapogenin extract from *M. sativa* (MSp) and 1% saponin extracts derived from *M. arborea* (MA), *M. polymorpha* cultivar Santiago (MPS), *M. polymorpha* cultivar Anglona (MPA), and *M. sativa* (MS), compared to those observed in 0.1% thiabendazole (TBZ) positive controls and in negative (1% DMSO and deionized water) controls.

As shown in Figure 2, compared to the negative controls, all tested samples at 1% concentration were able to strongly inhibit the development of donkey GIN eggs (*p* < 0.05), causing a reduction in egg hatching of ≥80%. 

MA and MS saponins significantly inhibited (*p* < 0.05) the development of donkey GIN eggs, causing a 100% reduction in egg hatching. Both these saponin mixtures showed an *in vitro* anthelmintic efficacy comparable (*p* < 0.05) to that of the reference drug 0.1% TBZ, for which an egg hatching reduction of about 97% (Figure 2) was observed. 

No statistical differences (*p* < 0.05) were also found when the activity of the reference drug was compared to those of MSp prosapogenins and MPA saponins, which were able to cause an egg hatching reduction of 90% and 88.7%, respectively (Figure 2). 

Among the tested compounds, only MPS saponins showed an *in vitro* anthelmintic activity significantly lower (*p* < 0.05) than that of all other tested compounds, as it caused an egg hatching reduction of 80% (Figure 2). 

Egg hatching reductions of 4% and 0% were instead observed in negative controls containing 1% DMSO and deionized water, respectively (Figure 2). 

Pooled coprocultures revealed a high prevalence of cyathostomins (>90%) in donkey pooled fecal samples, represented by species belonging to the genera *Cylicocyclus* and *Cylicostephanus*. The remaining 10% of identified nematodes included the species *Strongylus vulgaris, Strongylus equinus*, and *Triodontophorus* spp.

## 4. Discussion

In previous studies, the *in vitro* anthelmintic properties of saponins from different plant sources on eggs and larvae of goat gastrointestinal nematodes were reported [11,12]. Furthermore, the anthelmintic effect of *Combretum molle* extract against eggs and larvae of *Haemonchus contortus* was attributed to its saponin content [31]. Interestingly, an *in vivo* study showed that in sheep offered a diet containing 1.5% saponins from quillaja bark, the faecal egg count of GIN was reduced by 38.8% compared to animals receiving a diet without saponins [32]. 

In the present study, the evaluation of the *in vitro* anthelmintic activity on donkey GIN of saponins and prosapogenins from the different *Medicago* species was performed by using the EHT, a test considered valid for the evaluation of the *in vitro* efficacy of drugs on these nematodes [13,14,31]. Compared to negative controls, results from this study showed that all the *Medicago* saponin samples screened here were significantly effective in causing the inhibition of the development and hatching of donkey GIN eggs. Moreover, excluding MPS saponins, all the tested samples showed *in vitro* anthelmintic properties statistically comparable to that of the reference drug (0.1% TBZ). 

The mode of action of saponins on GIN eggs is not known. However, it is believed that their negative effects may be related with their ability to destabilize membranes and make them more permeable [11]. In this way, they may penetrate inside the eggs and destroy their contents, preventing the development of the nematode larva. Another hypothesis is that saponins may reduce the hatching rate of nematode eggs by interfering with the activity of enzymes responsible for hatching [11].

Moreover, the chemical structure is believed to play a key role in the bioactivity of the different saponins, and several studies were conducted to evaluate the specific role of aglycone as well as the nature and position of the sugars in the molecule [15,17,21,23,25,33]. However, these studies have provided contrasting results so far. In fact, in some cases there is evidence that sugar moieties may play a relevant role, while according to other studies, sapogenin moieties are more active [21]. Moreover, higher biological activity in general was reported for hederagenin and medicagenic acid glycosides, while lower activity was reported for echinocystic acid and zanhic acid [17,33]. 

Saponins from *Medicago* species are a complex mixture of triterpenic pentacyclic glycosides with medicagenic acid, hederagenin, zanhic acid, echinocystic acid, and soyasapogenol B as the dominant aglycones [15,17,18,19]. Sugar or sugar chains are attached at the triterpenic core of the molecule in selected positions to give monodesmosidic or bidesmosidic saponins [15]. 

The *in vitro* anthelmintic activity observed in this investigation can be related to the saponin/sapogenin composition of the different samples. Medicagenic acid and zanhic acid glycosides, found to be the most abundant constituents of MA, MS, and MSp samples, were the most active saponin mixtures, with bidesmosides (MA and MS) more active than monodesmosides (MSp). Echinocystic acid (MPA) and hederagenin (MPS) saponins resulted in lower activity.

## 5. Conclusions

Results obtained in this study showed for the first time that saponins from some *Medicago* plant species possess *in vitro* anthelmintic properties against donkey GIN. Among the tested saponins, MA, MS, and MSp samples were the most active, showing anthelminthic properties comparable to that of the control drug. Considering that the efficacy of an anthelmintic product is assured when it has at least 90% efficacy [34], these extracts should be considered as promising effective anthelmintic compounds on GIN of donkeys and, possibly, also of other animals. Therefore, further *in vitro* and *in vivo* studies aimed at evaluating their larvicidal and dose-dependent activity, and their toxicity and efficacy in treated animals are encouraged.
